# Four Surgical Cases of Medullary Carcinoma of the Colon

**DOI:** 10.70352/scrj.cr.25-0414

**Published:** 2025-09-13

**Authors:** Ayano Inao, Keisuke Noda, Tetsuro Tominaga, Toshio Shiraishi, Shintaro Hashimoto, Mariko Yamashita, Shoko Tei, Kazutaka Mochizuki, Nozomi Ueki, Takashi Nonaka, Keitaro Matsumoto

**Affiliations:** 1 Division of Surgical Oncology, Department of Surgery, Nagasaki University Graduate School of Biomedical Science, Nagasaki, Nagasaki, Japan; 2 Department of Pathology, Nagasaki University Graduate School of Biomedical Science, Nagasaki, Nagasaki, Japan; 3 Colorectal Surgery, Department of Surgery, Nagasaki University Hospital, Sakamoto, Nagasaki, Nagasaki, Japan; 4 Minimally Invasive Surgery Center, Nagasaki University Hospital, Nagasaki, Nagasaki, Japan

**Keywords:** medullary carcinoma, microsatellite instability, right colon

## Abstract

**INTRODUCTION:**

Medullary carcinoma of the colon was formerly classified as poorly differentiated adenocarcinoma. The prognosis is relatively good, with a high degree of microsatellite instability and a predilection for the right colon.

**CASE PRESENTATION:**

The mean age of the 4 patients was 69 years (range, 47–90 years), with 2 males and 2 females. Preoperative biopsy results showed 3 cases of moderately differentiated adenocarcinoma and 1 case of poorly differentiated adenocarcinoma. All cases were right-sided colon cancer, with 3 cases of ascending colon cancer and 1 case of transverse colon cancer. One case was classified as cT3, 2 as cT4a, and 1 as cT4b. One case was cN-negative, and 3 were cN-positive. No cases were cM-positive. The approach was laparoscopic in 3 cases and robot-assisted surgery in 1 case. Postoperative complications included postoperative ileus in 1 case. Pathological staging was Stage II in 3 cases and Stage III in 1 case. All cases showed high microsatellite instability, and no adjuvant therapy was administered. All patients remain under observation, with no recurrences identified at the time of writing.

**CONCLUSIONS:**

Medullary carcinoma is rare but has been increasing in recent years. This pathology shows characteristic histological features and immunophenotypes, and accurate diagnosis is important because of its effect on postoperative follow-up.

## Abbreviations

CA19-9
: carbohydrate antigen 19-9
CDX2
: caudal-related homeodomain protein 2
CEA
: carcinoembryonic antigen
CK
: cytokeratin
CS
: colonoscopy
dMMR
: deficient mismatch repair
HE
: hematoxylin and eosin
MLH 1
: MutL homolog 1
MSH
: mutS homolog
MSI
: microsatellite instability
MSI-H
: high microsatellite instability
PMS2
: postmeiotic segregation increased 2

## INTRODUCTION

Colonic medullary carcinoma is rare, accounting for 0.05%–0.08% of all colorectal cancers, and most cases exhibit an undifferentiated-like histological pattern.^1)^ This pathology is generally more common among middle-aged women and tends to occur in the right colon.^2)^ In addition, approximately 80% of cases exhibit high microsatellite instability (MSI-H) and deficient mismatch repair (dMMR). This tumor type is characterized by a better prognosis than typical undifferentiated carcinomas, and postoperative adjuvant chemotherapy with fluorouracil monotherapy is not recommended.^3,4)^ The histological concept of colorectal medullary carcinoma is relatively new, so reports remain limited. Here, we report 4 cases of colorectal medullary carcinoma treated surgically in our department, along with a review of the literature.

## CASE PRESENTATION

### Case 1

**Fig. 1:**
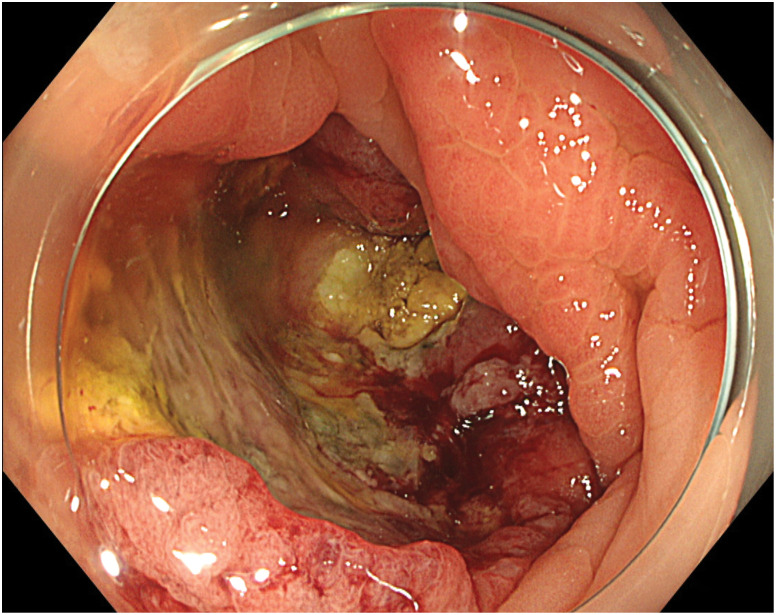
Lower gastrointestinal endoscopy. Endoscopy shows a type 2 tumor in the ascending colon.

**Fig. 2:**
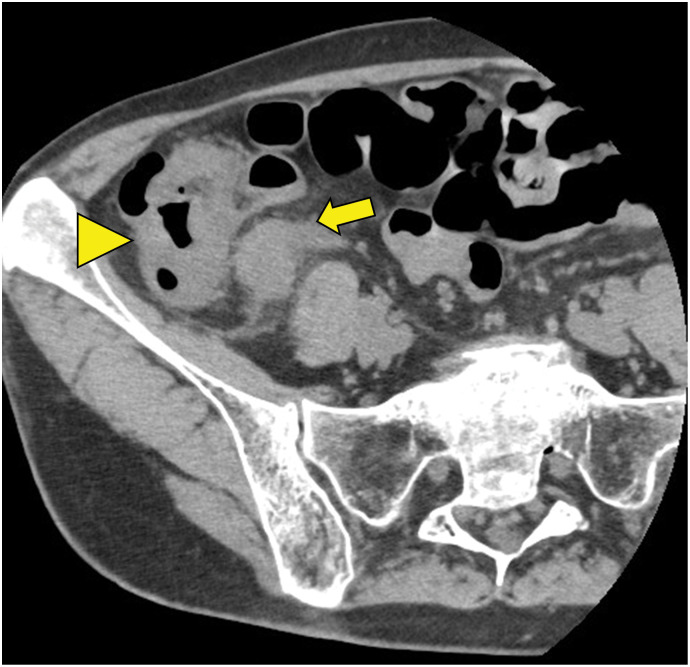
Contrast-enhanced CT. CT shows irregular wall thickening with contrast effect in the ascending colon (arrowhead). Regional lymphadenopathy is evident (arrow).

**Fig. 3:**
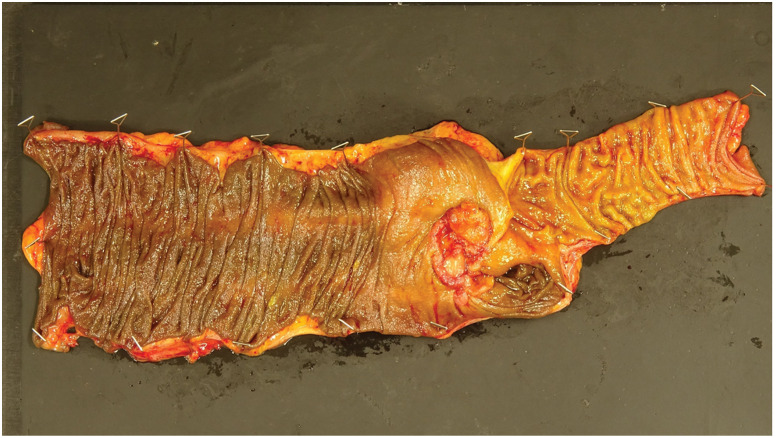
Examination of the resected specimen. Gross examination of the specimen reveals a type 2 lesion measuring 57 × 34 mm.

**Fig. 4:**
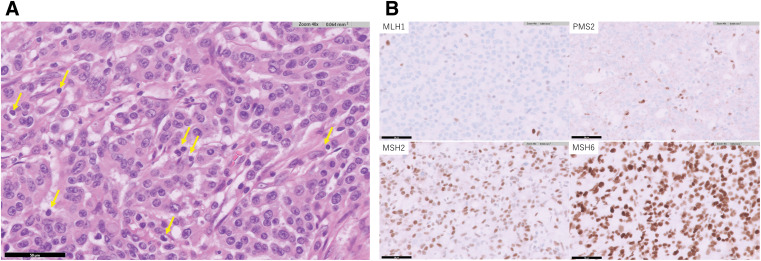
Pathological findings. (**A**) Histopathological examination reveals proliferating atypical epithelium with relatively distinct nucleoli and eosinophilic vacuoles, with prominent lymphocytic infiltration in the background (arrow). (**B**) Immunohistochemical staining shows MLH1(–), PMS2(–), MSH2(+), and MSH6(+), indicating dMMR. dMMR, deficient mismatch repair; MLH 1, MutL homolog 1; MSH, mutS homolog; PMS2, postmeiotic segregation increased 2

A 73-year-old woman showed a positive result on a fecal occult blood test and was referred to our institution from another facility, where colonoscopy (CS) had identified a type 2 lesion in the ascending colon (**Fig. 1**). The patient had a history of chronic renal failure. Biopsy led to a diagnosis of moderately differentiated adenocarcinoma. Tumor markers included carcinoembryonic antigen (CEA) at 1.3 n g/mL and carbohydrate antigen 19-9 (CA19-9) at 23.1 U/mL, both within normal ranges. Abdominal contrast-enhanced CT revealed marked wall thickening and mesenteric lymph node enlargement in the ascending colon (**Fig. 2**). The tumor was classified as cT4aN2aM0, Stage IIIC and the patient underwent laparoscopic ileocecal resection. The postoperative course was uneventful, and the patient was discharged on POD 8. Gross examination of the specimen revealed a type 2 lesion measuring 57 × 34 mm (**Fig. 3**). Histopathological examination revealed proliferating atypical epithelium with relatively distinct nucleoli and eosinophilic vacuoles, with prominent lymphocytic infiltration in the background (**Fig. 4A**). Immunohistochemical staining showed MutL homolog 1 (MLH1)(−), postmeiotic segregation increased 2 (PMS2)(−), mutS homolog 2 (MSH2)(+), MSH6(+), caudal-related homeodomain protein 2 (CDX2)(+), cytokeratin 20 (CK20) (−), and calretinin(−), indicating dMMR (**Fig. 4B**). A single metastasis was detected in a mesenteric lymph node, and medullary carcinoma, pT3N1aM0, pStage IIIB was diagnosed. Postoperative adjuvant therapy (XELOX for 3 months) was planned, but the patient declined treatment due to comorbidities and remains under observation. As of 3 months postoperatively, no obvious recurrence has been detected.

### Case 2

A 90-year-old woman underwent lower gastrointestinal endoscopy for further evaluation of chronic progressive anemia. A type 2 tumor was identified in the ascending colon. After a biopsy revealed poorly differentiated adenocarcinoma, she was referred to our department. Tumor marker levels included CEA at 1.6 ng/mL and CA19-9 at 10.9 U/mL, both within normal ranges. Abdominal contrast-enhanced CT revealed irregular wall thickening with contrast enhancement in the ascending colon near the ileum. No lymph nodes or distant metastases were detected, and the diagnosis was cT3N0M0, Stage IIA. Laparoscopic ileal resection was performed. The postoperative course was uneventful, and the patient was discharged on POD 11. Histopathological examination with hematoxylin and eosin (HE) staining revealed large nucleated cells with distinct nucleoli, eosinophilic cytoplasm, and a network-like dense focal distribution of cells with unclear polarity, as well as prominent lymphocytic infiltration in the stroma. Immunohistochemical staining showed MLH1(−), PMS2(+), MSH2(+), MSH6(+), CDX2(−), CK20(+), and calretinin(+). Based on these findings, medullary carcinoma, pT3N0M0, pStage IIA was diagnosed. As of 2 years and 6 months postoperatively, no obvious recurrence has been detected.

### Case 3

A 47-year-old man was referred to our department after a skin cancer follow-up revealed wall thickening in the ascending colon on abdominal CT. Biopsy of a sub-circumferential type 2 lesion in the ascending colon led to a diagnosis of differentiated adenocarcinoma. Tumor marker levels included CEA at 1.7 ng/mL and CA19-9 at 4.3 U/mL, both within normal ranges. Abdominal contrast-enhanced CT revealed irregular wall thickening with contrast enhancement and peritoneal invasion in the ascending colon. Enlarged regional lymph nodes were noted, but no distant metastasis was identified. The diagnosis was cT4b (peritoneum), N2aM0, cStage IIIC, and laparoscopic right hemicolectomy was performed. The postoperative course was uneventful and the patient was discharged on POD 7. Histopathological examination revealed well-differentiated, pleomorphic cells with solid and trabecular patterns. Immunohistochemical staining showed MLH1(+), PMS2(+), MSH2(−), MSH6(−), CDX2(+), CK20(−), and calretinin(−). Based on these findings, medullary carcinoma was diagnosed. No obvious lymph node metastases were detected, and the tumor was staged as pT4b (peritoneum), N0M0, pStage IIC. No adjuvant therapy was administered, and the patient is currently under observation. As of 1 year and 3 months after surgery, no obvious recurrence has been detected.

### Case 4

A 67-year-old man presented to another institution with constipation, where CS had identified a type 2 lesion in the hepatic flexure of the transverse colon. Biopsy led to a diagnosis of moderately differentiated adenocarcinoma. Tumor marker levels included CEA at 2.0 ng/mL and CA19-9 at 21.1 U/mL, both within normal ranges. Abdominal contrast-enhanced CT revealed enlarged mesenteric lymph nodes. The patient was diagnosed with cT4aN1bM0, cStage IIIC, and underwent robot-assisted right hemicolectomy. Postoperative intestinal obstruction developed but improved with conservative management, and the patient was discharged on POD 21. Histopathological examination with HE staining revealed proliferation of atypical epithelial cells with eosinophilic vacuoles. Immunohistochemical staining showed MLH1(−), PMS2(−), MSH2(+), MSH6(+), CDX2(+), CK20(−), and calretinin(−), indicating dMMR. No lymph node metastases were detected, and the diagnosis was characterized as medullary carcinoma, pT3N0M0, pStage IIA. No adjuvant therapy was administered, and the patient remains under follow-up. As of 4 months postoperatively, no obvious recurrence has been detected.

## DISCUSSION

Colonic medullary carcinoma is a rare tumor, with an incidence of 5–8 cases per 10000 individuals, as reported in a study covering the Surveillance, Epidemiology, and End Results database from 1973 to 2006.^5)^ In recent years, this pathology has shown a gradual increase in incidence, with Jabbal et al. reporting the average diagnosis rate for medullary carcinoma as 0.58% from 2004 to 2009, but increasing to 2.60% from 2010 to 2017.^6)^ This tumor is more common among older individuals, with an average age at diagnosis of 69 years and a female-to-male ratio of 2:1, and with women tending to be diagnosed at an older age than men.^7)^

The characteristics of the tumor include a higher incidence in the right colon, with an analysis of the National Cancer Database showing the following distribution: ascending colon, 34%; cecum, 31%; and transverse colon, 13%.^6)^ This distribution is thought to be associated with various factors such as the colonic microbiota, somatic mutation status, intestinal nutrient composition, and environmental factors, but the exact mechanisms remain unclear.^8)^ **Table 1** summarizes reports on medullary carcinoma of the colon from Japan, as identified from PubMed, including our cases.^9–14)^ The median age of the 12 cases was 72 years, with 83.3% (10/12) being female. The most common location was the ascending colon (9 cases, 75.0%), followed by the transverse colon (3 cases, 25.0%). The stage distribution was as follows: Stage II in 7 cases (58.3%), Stage III in 4 cases (33.3%), and Stage I in 1 case (8.3%). All patients did not receive adjuvant therapy. One case died 3 months postoperatively, but all other cases with reported outcomes remained alive at the final follow-up. However, the observation period in several case reports was too short to conclusively state that no recurrence had occurred. Further investigation, including of the long-term prognosis, is therefore necessary.

**Table 1:** Japanese case reports of medullary carcinoma of the colon

Authors	Age (years)	Sex	Location	Pathological stage	MSI-H/dMMR	Adjuvant chemotherapy	Prognosis
Tatsuta et al.^9)^	70	Male	Ascending colon	IIIC	Yes	No	10 months; alive
Saeki et al.^10)^	79	Female	Transverse colon	IIA	Yes	UFT/UZEL 6 months	18 months; alive
Morimoto et al.^11)^	93	Female	Ascending colon	IIA	Yes	No	Unknown
	91	Female	Ascending colon	IIA	Unknown	No	Unknown
	65	Female	Ascending colon	IIIB	Unknown	No	Unknown
Kobayashi et al.^12)^	59	Female	Transverse colon	I	Unknown	No	5 months; alive
Tamai et al.^13)^	87	Female	Ascending colon	IIB	Yes	No	3 months; alive
Wakasugi et al.^14)^	72	Female	Ascending colon	IIIB	Yes	No	3 months; dead
Our cases	73	Female	Ascending colon	IIIB	Yes	No	3 months; alive
	90	Female	Ascending colon	IIA	Yes	No	12 months; alive
	47	Male	Ascending colon	IIC	Yes	No	15 months; alive
	67	Female	Transverse colon	IIA	Yes	No	4 months; alive

dMMR, deficient mismatch repair; MSI-H, high microsatellite instability

Pathologically, medullary carcinoma is characterized by sheet-like structures of malignant cells, cystic nuclei, prominent nucleoli, abundant cytoplasm, and marked infiltration of intraepithelial lymphocytes.^15)^ Immunohistochemistry is useful for diagnosis, with specific stains indicating intestinal differentiation (CDX2 or CK20) typically yielding negative results, whereas calretinin and cytokeratin 7 (CK7) are often positive.^16)^ Medullary carcinoma shares similar histological features with undifferentiated adenocarcinoma and poorly differentiated adenocarcinoma, but is reported to show a relatively favorable prognosis.^5,17,18)^ Compared to the 1- and 2-year survival rates of 70.5% and 58.4% reported for undifferentiated adenocarcinoma, medullary carcinoma shows better outcomes, at 92.7% and 73.8%, respectively.^5)^ Medullary carcinoma is generally characterized by minimal perineural invasion, lymph node metastasis, and distant metastasis.^6)^ This may be one of the factors contributing to the improved survival rate of patients with medullary carcinoma. In this study, immunohistochemical staining revealed negative results for CK20 in 3 of the 4 cases (75%), negative results for CDX2 in 2 cases (50%), positive results for CDX2 in 2 cases (50%), and positive results for calretinin in all cases (100%), supporting previous reports.

Another characteristic of medullary carcinoma is that 60%–80% of cases are associated with dMMR proteins and MSI-H.^16,19)^ MSI-H tumors are considered to have a relatively favorable prognosis.^20)^ MSI-H tumors are highly mutated tumors that produce a large number of neoantigens. As a result, these tumors are more easily recognized and targeted by the immune system, and by T cells in particular. In fact, MSI-H tumors are known to be associated with a higher number of tumor-infiltrating lymphocytes, which is considered one reason for the favorable prognosis. Immunohistochemical patterns differ between medullary carcinoma and other poorly differentiated adenocarcinomas.^8)^ Medullary carcinoma often shows loss of MLH1 and PMS2, whereas poorly differentiated adenocarcinomas typically do not. Medullary carcinoma is often CDX2/CK20-negative in colorectal cancer, including typical poorly differentiated adenocarcinomas. In addition, medullary carcinoma is characterized by strong positivity for CD3 and CD8, which indicates lymphocytic infiltration. Many previous studies have detected deletions of MLH1 and PMS2, and confirmation by immunohistochemistry is an important diagnostic criterion.^16,19)^

In this study, all cases were dMMR/MSI-H, with MLH1 deletion in 3 cases and PMS2 deletion in 2 cases, both of which were useful for diagnosis.

MSI-H colorectal cancer exhibits resistance to 5-fluorouracil, and no consensus has been established regarding oxaliplatin.^21–23)^ As described above, medullary-type colon cancer, particularly right-sided colon cancer, is highly likely to be MSI-H. Especially for Stage III cases, decisions regarding adjuvant chemotherapy should be made based on a comprehensive evaluation, including age, comorbidities, and pathological risk factors. The 4 cases in this study comprised 3 cases of Stage II and 1 case of Stage III. No adjuvant therapy was administered, and all cases are currently disease-free.

Previous reports have indicated that in endoscopic biopsies of medullary carcinoma of the colon, the majority of cases are initially diagnosed as undifferentiated adenocarcinoma. Upon re-evaluation of surgically resected specimens, the diagnosis is then confirmed to be medullary carcinoma.^24)^ Medullary carcinoma shows a high proportion of MSI-H, which is an important factor in determining postoperative treatment strategies (such as adjuvant chemotherapy). In cases where medullary carcinoma is suspected, definitive diagnosis including immunohistochemistry and microsatellite instability (MSI)/MMR testing is crucial to determining the optimal treatment strategy.

## CONCLUSIONS

Medullary carcinoma is rare but has been increasing in recent years. Accurate diagnosis based on the characteristic histological features and immunophenotypes is important because of its impact on postoperative follow-up.

## DECLARATIONS

### Availability of data and materials

The data that support the findings of this study are available from the corresponding author upon reasonable request.

## References

[1] Pyo JS, Sohn JH, Kang G. Medullary carcinoma in the colorectum: a systematic review and meta-analysis. Hum Pathol 2016; 53: 91–6. 27001432 10.1016/j.humpath.2016.02.018

[2] Martinotti M, Cirillo F, Ungari M, et al. Microsatellite instability in medullary carcinoma of the colon. Rare Tumors 2016; 9: 23–5. 10.4081/rt.2017.6541 PMC5391516 28458789

[3] Wick MR, Vitsky JL, Ritter JH, et al. Sporadic medullary carcinoma of the colon: a clinicopathologic comparison with nonhereditary poorly differentiated enteric-type adenocarcinoma and neuroendocrine colorectal carcinoma. Am J Clin Pathol 2005; 123: 56–65. 15762280

[4] Hashiguchi Y, Muro K, Saito Y, et al. Japanese Society for Cancer of the Colon and Rectum (JSCCR) guidelines 2019 for the treatment of colorectal cancer. Int J Clin Oncol 2020; 25: 1–42. 31203527 10.1007/s10147-019-01485-z PMC6946738

[5] Thirunavukarasu P, Sathaiah M, Singla S, et al. Medullary carcinoma of the large intestine: a population based analysis. Int J Oncol 2010; 37: 901–7. 20811712 10.3892/ijo_00000741 PMC4127912

[6] Jabbal IS, Nagarajan A, Rivera C, et al. Medullary carcinoma of the colon: a comprehensive analysis of the National Cancer Database. Surg Oncol 2022; 45: 101856. 36446307 10.1016/j.suronc.2022.101856

[7] Cunningham J, Kantekure K, Saif MW. Medullary carcinoma of the colon: a case series and review of the literature. In Vivo 2014; 28:311–4. 24815832

[8] Winn B, Tavares R, Fanion J, et al. Differentiating the undifferentiated: immunohistochemical profile of medullary carcinoma of the colon with an emphasis on intestinal differentiation. Hum Pathol 2009; 40: 398–404. 18992917 10.1016/j.humpath.2008.08.014 PMC2657293

[9] Tatsuta K, Sakata M, Iwaizumi M, et al. Mismatch repair proteins immunohistochemical null phenotype in colon medullary carcinoma. Clin J Gastroenterol 2021; 14: 1448–52. 34279804 10.1007/s12328-021-01484-6

[10] Saeki S, Maeda Y, Nishida Y, et al. A case of medullary carcinoma of the colon. Gan To Kagaku Ryoho 2023; 50: 511–3. (in Japanese) 37066471

[11] Morimoto Y, Takaoka M, Monobe Y, et al. Three cases of colon medullary carcinoma in our institution. Gan To Kagaku Ryoho 2021; 48: 967–9. (in Japanese) 34267038

[12] Kobayashi T, Ogawa S, Kurioka H. A case of medullary carcinoma of transverse colon which resected by laparoscopic colectomy. Gan To Kagaku Ryoho 2021; 48: 288–90. (in Japanese) 33597384

[13] Tamai K, Okamura S, Kitahara T, et al. A case of medullary carcinoma of the colon with poor prognosis. Gan To Kagaku Ryoho 2020; 47: 637–9. (in Japanese) 32389968

[14] Wakasugi M, Kono H, Yasuhara Y, et al. A resected case of medullary carcinoma of the ascending colon followed by infarction of the greater omentum mimicking anastomotic leakage. Int J Surg Case Rep 2017; 41: 456–60. 29546016 10.1016/j.ijscr.2017.11.027 PMC5712804

[15] Nagtegaal ID, Odze RD, Klimstra D, et al. The 2019 WHO classification of tumours of the digestive system. Histopathology 2020; 76: 182–8. 31433515 10.1111/his.13975 PMC7003895

[16] Gómez-Álvarez MA, Lino-Silva LS, Salcedo-Hernández RA, et al. Medullary colonic carcinoma with microsatellite instability has lower survival compared with conventional colonic adenocarcinoma with microsatellite instability. Prz Gastroenterol 2017; 12: 208–14. 29123583 10.5114/pg.2016.64740 PMC5672702

[17] Rüschoff J, Dietmaier W, Lüttges J, et al. Poorly differentiated colonic adenocarcinoma, medullary type: clinical, phenotypic, and molecular characteristics. Am J Pathol 1997; 150: 1815–25. 9137104 PMC1858211

[18] Lanza G, Gafà R, Matteuzzi M, et al. Medullary-type poorly differentiated adenocarcinoma of the large bowel: a distinct clinicopathologic entity characterized by microsatellite instability and improved survival. J Clin Oncol 1999; 17: 2429–38. 10561306 10.1200/JCO.1999.17.8.2429

[19] Lin F, Shi J, Zhu S, et al. Cadherin-17 and SATB2 are sensitive and specific immunomarkers for medullary carcinoma of the large intestine. Arch Pathol Lab Med 2014; 138: 1015–26. 24437456 10.5858/arpa.2013-0452-OA

[20] Fan WX, Su F, Zhang Y, et al. Oncological characteristics, treatments and prognostic outcomes in MMR-deficient colorectal cancer. Biomark Res 2024; 12: 89. 39183366 10.1186/s40364-024-00640-7 PMC11346251

[21] Ribic CM, Sargent DJ, Moore MJ, et al. Tumor microsatellite-instability status as a predictor of benefit from fluorouracil-based adjuvant chemotherapy for colon cancer. N Engl J Med 2003; 349: 247–57. 12867608 10.1056/NEJMoa022289 PMC3584639

[22] Kim GP, Colangelo LH, Wieand HS, et al. Prognostic and predictive roles of high-degree microsatellite instability in colon cancer: a National Cancer Institute-National Surgical Adjuvant Breast and Bowel Project Collaborative Study. J Clin Oncol 2007; 25: 767–72. 17228023 10.1200/JCO.2006.05.8172

[23] Barratt PL, Seymour MT, Stenning SP, et al. DNA markers predicting benefit from adjuvant fluorouracil in patients with colon cancer: a molecular study. Lancet 2002; 360: 1381–91. 12423985 10.1016/s0140-6736(02)11402-4

[24] Fatima Z, Sharma P, Youssef B, et al. Medullary carcinoma of the colon: a histopathologic challenge. Cureus 2021; 13: e15831. 34327072 10.7759/cureus.15831 PMC8301270

